# Real-world effectiveness and safety of Baloxavir Marboxil or Oseltamivir in outpatients with uncomplicated influenza A: an ambispective, observational, multi-center study

**DOI:** 10.3389/fmicb.2024.1428095

**Published:** 2024-07-23

**Authors:** Jianpeng Cai, Hongyu Wang, Xiaoting Ye, Shengjia Lu, Zhili Tan, Zhonghua Li, Dan Lin, Jiancheng Qian, Xiaoxian Lu, Jiaolong Wan, Jie Wang, Jingwen Ai, Yonglan Pu, Lihong Qu, Sen Wang

**Affiliations:** ^1^Department of Infectious Diseases, Shanghai Key Laboratory of Infectious Diseases and Biosafety Emergency Response, National Medical Center for Infectious Diseases, Huashan Hospital, Shanghai Medical College, Fudan University, Shanghai, China; ^2^Department of Infectious Diseases, The Third Affiliated Hospital of Wenzhou Medical University, Wenzhou, China; ^3^Department of Infectious Diseases, Tongde Hospital of Zhejiang Province, Hangzhou, China; ^4^Department of Infectious Diseases, East Hospital Affiliated to Tongji University, Shanghai, China; ^5^Department of Infectious Disease, Taicang First People's Hospital, Suzhou, China; ^6^Department of Infectious Disease, People's Hospital (Jiangnan University Medical Center), Wuxi, China; ^7^Department of Infectious Diseases, East Hospital Affiliated to Tongji University, Shanghai, China; ^8^Shanghai Sci-Tech InnoCenter for Infection and Immunity, Shanghai, China

**Keywords:** influenza, Baloxavir Marboxil, Oseltamivir, real-world study, effectiveness

## Abstract

**Introduction:**

Baloxavir Marboxil is a per oral small-molecule antiviral for the treatment of influenza. While the efficacy and safety of Baloxavir Marboxil have been thoroughly characterized across an extensive clinical trial, studies on the effectiveness of Baloxavir Marboxil in a real-world setting are still scarce.

**Methods:**

We conducted an ambispective, observational, multi-center study that enrolled uncomplicated in-fluenza outpatients treated with Baloxavir Marboxil or Oseltamivir in East China. The primary endpoint was time from treatment to alleviation of all influenza symptoms (TTAIS). The secondary endpoints included time from treatment to alleviation of fever (TTAF) and household transmission during the duration of influenza.

**Results:**

A total of 509 patients were enrolled. The median TTAIS in the Baloxavir Marboxil group and the Oseltamivir group was 28.0  h (IQR, 20.0 to 50.0) and 48.0  h (IQR, 30.0 to 67.0), respectively. The median TTAF in the Baloxavir Marboxil group and the Oseltamivir group was 18  h (IQR, 10.0–24.0) and 30.0  h (IQR, 19.0–48.0). In the COX multivariable analysis, Baloxavir Marboxil reduced the duration of influenza symptoms (HR  =  1.36 [95%CI:1.12–1.64], *p* =  0.002) and the duration of fever (HR  =  1.93 [95%CI:1.48–2.52], *p* < 0.001) compared to Oseltamivir. When antiviral drugs were given within 12–48  h after symptom onset, the Baloxavir Marboxil group had a significantly shorter TTAIS compared to the Oseltamivir group. There was no significant difference in the rate of adverse events between the two group (*p* = 0.555).

**Discussion:**

Baloxavir Marboxil was superior to Oseltamivir in alleviating influenza symptoms in outpatients with uncomplicated influenza. Our findings suggested that compared to Oseltamivir, Baloxavir Marboxil might be more appropriate for patients with influenza 12– 48 h after symptom onset.

## Introduction

Influenza is one of the main causes of the acute respiratory infection (ARI), which causes epidemic every year and pandemic at varied interval before COVID-19 broke out (Girit et al., 2018). During the past 100 years, there was 4 pandemics including H1N1 at 1918, H2N2 during 1957–1958, H3N2 during 1968–1969, and H1N1 in 2009 (World Health Organization, 2023a), which brought traumatic losses to human beings. Besides these pandemics, influenza still causes epidemics every year all around the world. According to Chinese Center for Disease Control and Prevention (Prevention, 2021), there was 3,538,213 cases reported in 2019, with an consultation rate of 253.36/10,000. Between 2010 and 2015, 88,100 excess respiratory deaths were caused by influenza in China each year (Li et al., 2019).

Comprehensive strategies should be taken to prevent influenza endemic (World Health Organization, 2023b), including active surveillance on the incidence and variants, annual vaccination among high-risk populations, early diagnosis for influenza including serial point-of-care detections, as well as administration of effective antiviral drugs. Oseltamivir is a widely available oral antiviral for influenza, which acts as a competitive inhibitor of the neuraminidase enzyme and has shown effective performance in reducing symptom duration, illness progression and also prophylactic prevention (Widmer et al., 2010; Yu et al., 2010; Jaiswal et al., 2020). However, the appearance of Oseltamivir-resistant H1N1pdm09 since 2009 has posed a threat to the public health (Hurt et al., 2012).

Baloxavir Marboxil was a per oral small-molecule antiviral with different antiviral mechanism as the prodrug of the selective PA inhibitor S-033447, which has shown nanomolar antiviral activity against influenza A and B viruses including strains resistant to current antiviral agents (Noshi et al., 2018; Taniguchi et al., 2019). A series of randomized, double-blind controlled phase-3 clinical trials (CAPSTONE-1 and CAPSTONE-2) has proved the clinical efficacy of shortening symptom duration, reducing viral load, preventing illness progression among high-risk population, post-exposure prophylaxis and satisfying safety of Baloxavir Marboxil compared to both placebo and Oseltamivir (Hayden et al., 2018; Baker et al., 2020; Ikematsu et al., 2020; Ison et al., 2020). In a real-world study in Japan, Baloxavir may reduce hospitalization compared with oseltamivir and zanamivir (Komeda et al., 2021a). However, according to our best knowledge, there is currently no efficacy and safety data of Baloxavir yet among Chinese influenza patients. Besides, whether a single-dose administration of Baloxavir Marboxil can reduce household transmission between patients and close contact remains to be further investigated.

**Figure 1:**
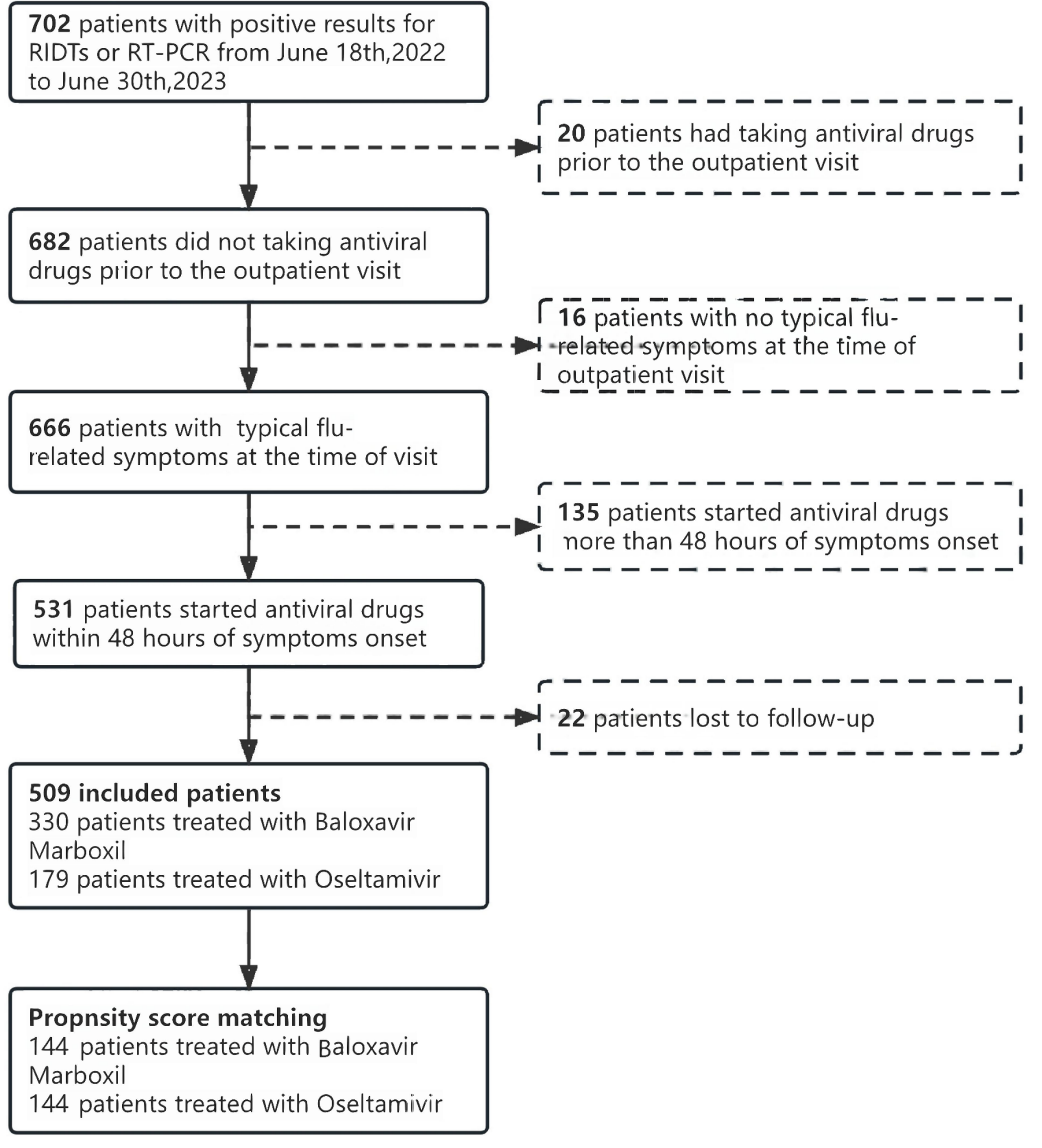
Flow chart.

To figure out the real-world effectiveness of Baloxavir Marboxil among uncomplicated Influenza compared to Oseltamivir, we conducted this ambispective, real-world cohort study to evaluate the symptom alleviation and transmission reduction potential for preventing household transmission after administration of Baloxavir Marboxil to infected patients (see Figure 1).

## Methods

### Study design

This was a multicenter real-world ambispective cohort study conducted in outpatient fever clinics in East China, including Shanghai, Taicang, Ruian, Changshu and Hangzhou between 2022.06 and 2023.06. Patients presented with fever or influenza-like symptoms would undergo test for influenza. As Baloxavir’s indication was restricted among uncomplicated influenza for patients aged 12 years old and above by the time this study was designed (indication for younger children was not approved until March 2023 in China), those with complicated influenza or < 12 years of age would be excluded. The detailed inclusion criteria were: (1) ≥ 12 years old; (2) positive results for Rapid influenza diagnostic tests (RIDTs) or reverse-transcription polymerase chain reaction (RT-PCR); (3) The time interval between the onset of symptoms and screening was ≤48 h; (4) present with fever (axillary temperature ≥ 37.5°C or tympanic temperature of ≥38.5°C) or one influenza-like symptoms including cough, sore throat, nasal congestion or runny nose, headache, chills, muscle or joint pain, and fatigue; (5) In the retrospective study, patients contracted influenza within the 3 months prior to the start of the study, specifically from June 2022 to September 2022. The excluding criteria were: (1) pregnant or positive HCG result before drug administration; (2) known hypersensitivity to Baloxavir Marboxil or drug excipients; (3) severe influenza infection requiring hospitalization; (4) any other conditions that physician considered unsuitable for enrollment. The patients at high risk for influenza-associated complications are those with at least one of the following criteria: chronic lung disease; cardiovascular disease; endocrine disease; renal disease; hepatic disease; neurologic disease; malignant tumor; immunosuppression status and age ≥ 65 years (China, 2020). According to doctors’ prescriptions, patients were divided into Baloxavir Marboxil and Oseltamivir groups. Some patients received routine clinical symptomatic treatment, including antipyretic-analgesics agents, and antitussives/expectorants agents. Oral informed consent was obtained from all participants or their legal guardians.

### Data collection and follow-up

From June 18^th^, 2022 to June 30^th^, 2023, after receiving consents from the participants or their guardians, demographic information, disease history, vital signs, disease condition and prescribed drugs were collected for each participant at the baseline. In the prospective cohort, researchers would contact the participants over the phone at day 3, 7 after enrollment to follow up for symptoms, complications, suspected adverse effect, household transmission related to influenza. In the retrospective cohort, researchers would contact the participants over the phone to collect information about symptoms, complications, suspected adverse effect, and household transmission related to influenza within 7 days after taking the antiviral drugs.

### Effectiveness

The primary endpoint was time from treatment to alleviation of all influenza symptoms (TTAIS), including fever, cough, nasal congestion or runny nose, headache, chills, muscle or joint pain, and fatigue. The secondary endpoints included time from treatment to alleviation of fever (TTAF) and household transmission during the duration of the 7-day follow-up. The alleviation of fever was defined as axillary temperature of <37.5°C or tympanic temperature of <38.5°C. Body temperature and whether other influenza symptoms were alleviated came from patients’ self-report.

### Safety

The safety endpoint was the occurrence of suspected the adverse events during and post drug administration, including overall and incidence of each adverse events, serious adverse events, and discontinuation of drug usage due to adverse events. The definition of adverse events: any adverse medical event self-reported by the participants within 7 days of taking antiviral medication.

### Household influenza transmission

The transmission rate of household influenza was the percentage of patients reporting the presence of suspected or confirmed influenza in the household during the follow-up period after excluding subjects with other influenza-infected families present in the household at the time of enrollment. Suspected influenza-infected family members were defined as having influenza symptoms (including fever, cough, nasal obstruction, headache, chills, muscle or joint pain, and fatigue) but not tested for RIDTs or RT-PCR. Confirmed influenza-infected family members were defined as having influenza symptoms and positive test for RIDTs or RT-PCR. The positive test for RIDTs or RT-PCR was self-reported by patients.

### Statistical analysis

SPSS 22.0, GraphPad Prism 8 and R software 4.2.2 were applied for statistical analysis. Continuous variables were expressed as median (Range) or mean (Standard Deviation, SD) and compared with the non-parametric test. Categorical variables were expressed as number (%) and compared by the χ^2^ test or Fisher’s exact tests. Propensity Score Matching was used to balance the age, gender, high risk of developing influenza-associated complication, vaccination status, use of analgesic- antipyretic drugs and antitussive/expectorant agents among the two groups. Cases were matched 1:1 with a caliper size of 0.02. The primary analysis compared the TTAIS between the two groups using Kaplan–Meier Curve, Log-rank test and two-stage way (Qiu and Sheng, 2007) when the curves crossed. To further confirm the robustness of the effectiveness of Baloxavir Marboxil and Oseltamivir, hazard ratio (HR) and 95% confidence interval were calculated by Cox regression with adjustment for the following potential confounders: sex (male/female), age (continuous), influenza vaccination status (yes/no), time from symptom onset to antiviral drugs (<12 h/>12 h and < 24 h/>24 h and < 36 h/>36 h and < 48 h). A two-sided *p* < 0.05 was considered statistically significant.

## Results

A total of 509 influenza patients participated in this study, with 118 retrospectively included and 391 prospectively included (Supplementary Table S1). The median age of the participants was 34 years (Table 1). Six participants received influenza vaccination within 6 months, and 50 participants had high-risk factors for influenza-associated complications. Among them, 330 received treatment with Baloxavir Marboxil, and 179 received treatment with Oseltamivir. The Baloxavir Marboxil group and the Oseltamivir group showed no significant differences in age, gender, or influenza vaccination history. However, the proportion of participants with high-risk factors for influenza-associated complications was higher in the Baloxavir Marboxil group than in the Oseltamivir group (11.82% vs. 6.15%, *p* = 0.04). After 1:1 propensity score matching, there were 144 participants in each group, and the matched groups showed no significant differences in age, gender, influenza vaccination history, symptomatic treatments or high-risk factors for influenza-associated complications (Table 1).

**Table 1:** Demographic and clinical characteristic of participants.

Overall					1:1 matched sample		
Characteristics	Total *n* = 509	Baloxavir Marboxil *n* = 330	Oseltamivir *n* = 179	*p* value	Baloxavir Marboxil *n* = 144	Oseltamivir *n* = 144	*p* value
Gender, n(%)				0.482			0.637
Male	261 (51.28)	173 (52.42)	88 (49.16)		73 (50.69)	69 (47.92)	
Female	248 (48.72)	157 (47.58)	91 (50.84)		71 (49.31)	75 (52.08)	
Age, mean ± SD [Median, IQR] (years)	36.7 ± 14.6 [34, 26–44]	37.3 ± 14.9 [35, 27–45]	35.4 ± 13.7 [33, 26–41]	0.128	35.8 ± 13.9[33.5,26.5–41.5]	36.3 ± 14.2 [33,26–41]	0.731
Time from symptom onset to antiviral drugs, n (%)				0.112			0.111
≤12 h	83 (16.31)	53 (16.06)	30 (16.76)		22 (15.28)	24 (16.67)	
>12 to ≤24 h	153 (30.06)	109 (33.03)	44 (24.58)		52 (36.11)	34 (23.61)	
>24 to ≤36 h	212 (41.65)	135 (40.91)	77 (43.02)		54 (37.50)	62 (43.06)	
>36 to ≤48 h	61 (11.98)	33 (11.98)	28 (15.64)		16 (11.11)	24 (16.67)	
Influenza vaccination, *n* (%)				0.095			1.000
Yes	6 (1.18)	6 (1.82)	0 (0)		0 (0)	0 (0)	
No	503 (98.82)	324 (98.18)	179 (100)		144 (100)	144 (100)	
Comorbidities, *n* (%)							
Hypertension	23 (4.52)	17 (5.15)	6 (3.35)	0.351	3 (2.08)	5 (3.47)	0.723
Diabetes	10 (1.96)	10 (3.03)	0 (0)	0.019	2 (1.39)	0 (0)	0.498
Cardiovascular disease (except Hypertension)	8 (1.57)	7 (2.12)	1 (0.56)	0.176	0 (100)	1 (0.69)	1.000
Endocrine diseases (except Diabetes)	5 (0.98)	3 (0.91)	2 (1.12)	1.000	1 (0.69)	2 (1.39)	1.000
Lung disease	4 (0.79)	3 (0.91)	1 (0.56)	1.000	1 (0.69)	1 (0.69)	1.000
Hepatic disease	4 (0.79)	2 (0.61)	2 (1.12)	0.616	1 (0.69)	2 (1.39)	1.000
Hematologic disease	2 (0.39)	1 (0.30)	1 (0.56)	1.000	1 (0.69)	1 (0.69)	1.000
Cancer	1 (0.20)	1 (0.30)	0 (0)	1.000	0 (0)	0 (0)	1.000
High risk of developing influenza-associated complications, *n* (%)	50 (9.82)	39 (11.82)	11 (6.15)	0.040	11 (7.64)	11 (7.64)	1.000
Influenza symptoms, *n* (%)							
Fever	275 (54.03)	180 (54.55)	95 (53.07)	0.750	67 (46.53)	74 (51.39)	0.409
Cough	367 (72.10)	223 (67.58)	144 (80.45)	0.002	97 (67.36)	119 (82.64)	0.003
Sore throat	283 (55.60)	194 (58.79)	89 (49.72)	0.049	79 (54.86)	71 (49.31)	0.345
Nasal congestion or runny nose	165 (32.42)	119 (36.06)	46 (25.70)	0.017	52 (36.11)	39 (27.08)	0.099
Chills	147 (28.88)	100 (30.30)	47 (26.26)	0.336	39 (27.08)	37 (25.69)	0.789
Headache	237 (46.56)	157 (47.58)	80 (44.69)	0.534	71 (49.31)	71 (49.31)	1.000
Muscle or joint pain	258 (50.69)	161 (48.79)	97 (54.19)	0.244	72 (50)	81 (56.25)	0.288
Fatigue	188 (36.94)	126 (38.18)	62 (34.64)	0.429	50 (34.72)	54 (37.5)	0.624
Analgesic-antipyretic	364 (71.51)	253 (76.67)	111 (62.01)	<0.001	104 (72.22)	106 (73.61)	0.791
Antitussive and expectorant agents	271 (53.24)	182 (55.15)	89 (49.72)	0.241	78 (54.17)	80 (55.56)	0.813
Duration of symptoms, mean ± SD [Median, IQR] (hour)	41.6 ± 27.5 [37, 22–60]	37.8 ± 28.7 [28, 20–50]	48.6 ± 23.7 [48, 30–67]	<0.001	39.8 ± 34.0 [28,16–54.5]	49.1 ± 23.8 [48,30.5–68]	0.007
Duration of fever, mean ± SD [Median, IQR] (hour)	26.8 ± 21.3 [21, 12–36]	21.8 ± 18.2 [18, 10–24]	36.1 ± 23.3 [30, 19–48]	<0.001	18.5 ± 13.7 [15,9–24]	36.5 ± 24.9 [32,20–48]	<0.001

**Figure 2:**
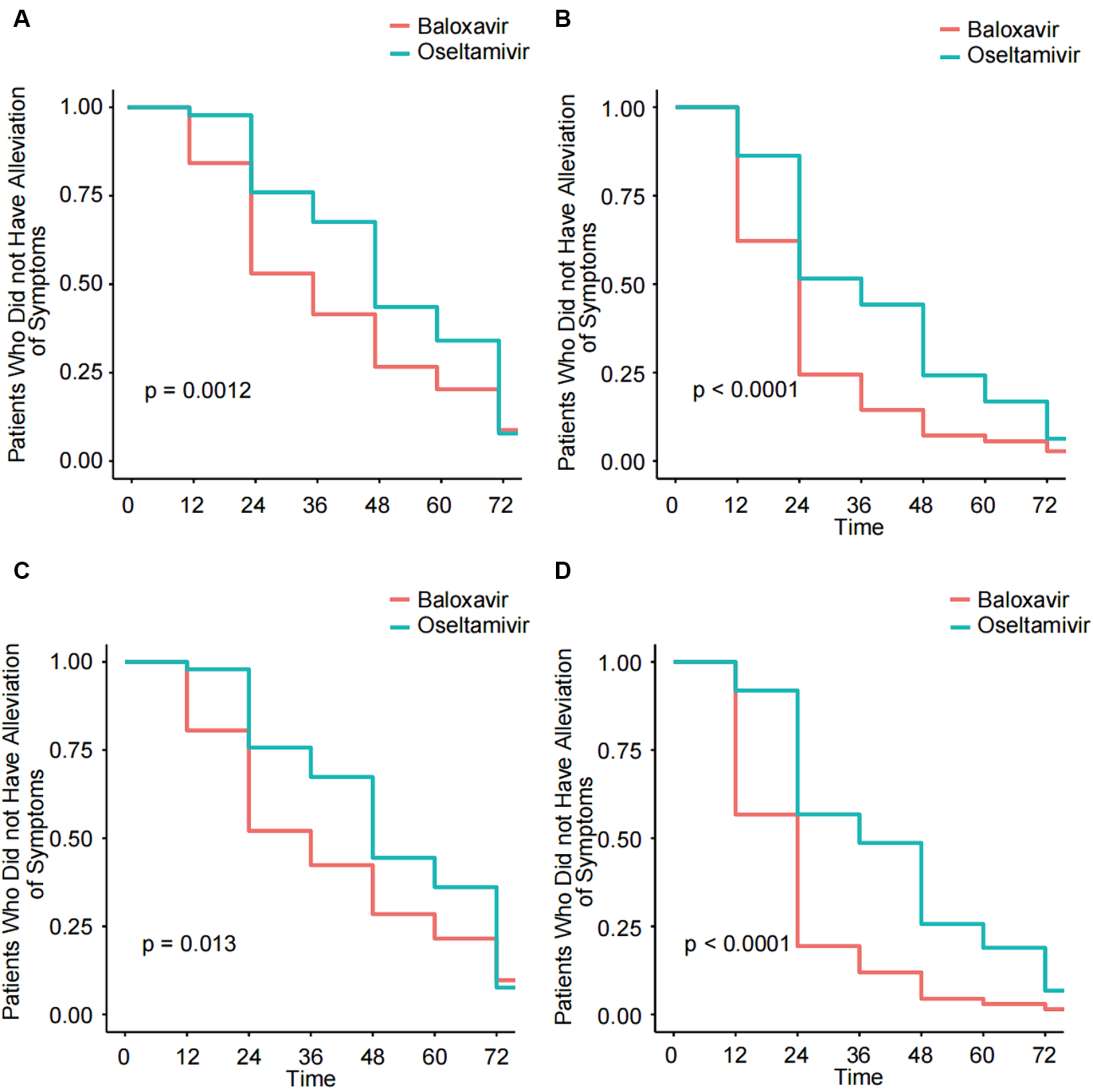
Kaplan–Meier analysis of the TTAIS and the TTAF in the Baloxavir Marboxil group and the Oseltamivir group before and after 1:1 matched. **(A)** Kaplan–Meier analysis of the TTAIS in the Baloxavir Marboxil group and the Oseltamivir group. **(B)** Kaplan–Meier analysis of the TTAF in the Baloxavir Marboxil group and the Oseltamivir group. **(C)** Kaplan–Meier analysis of the TTAIS in the Baloxavir Marboxil group and the Oseltamivir group after 1:1 matched. **(D)** Kaplan–Meier analysis of the TTAF in the Baloxavir Marboxil group and the Oseltamivir group after 1:1 matched.

First, we analyzed the TTAIS and the TTAF in the Baloxavir Marboxil and Oseltamivir groups. In this study, the median TTAIS in the Baloxavir Marboxil group was significantly lower than in the Oseltamivir group (28.0 h [20–50] and 48.0 h [30–67], *p* < 0.001). Similarly, the median TTAF in the Baloxavir Marboxil group was significantly lower than in the Oseltamivir group (18 h [10–24] and 30 h [19–48], *p* < 0.001). After propensity score matching, the median TTAIS and the median TTAF in the Baloxavir Marboxil group remained significantly lower than in the Oseltamivir group (Table 1). The same results were observed in the KM curves (Figures 2A–D). Additionally, the duration of cough, sore throat, chills, headache, muscle or joint pain, and fatigue in the Baloxavir Marboxil group were shorter than in the Oseltamivir group (Supplementary Figure S1). After propensity score matching, the same results were observed (Supplementary Figure S2). To confirm the robustness of the results, we used a multivariate COX proportional hazards regression model for further evaluation. In the COX multivariable analysis, compared to Oseltamivir, Baloxavir Marboxil could significantly reduce the TTAIS (HR = 1.35 [95%CI:1.12–1.64], *p* = 0.002), and the TTAF (HR = 1.95 [95%CI:1.49–2.55], *p* < 0.001) (Table 2). We assessed the effect of symptomatic treatments on symptoms. Compared to using Baloxavir Marboxil or Oseltamivir alone, combining symptomatic treatments did not significantly reduce either the TTAIS or the TTAF (Supplementary Figure S3).

**Table 2:** Multivariable-adjusted hazard ratios (aHRs) for the TTAIS and TTAF in participants.

	TTAIS (h)		TTAF (h)	
Characteristics	aHRs (95%CI)	*p* values	aHRs (95%CI)	*p* value
Gender				
Female	Reference	–	Reference	–
Male	0.97 (0.81–1.16)	0.762	0.86 (0.68–1.10)	0.226
Age group(years)	0.99 (0.98–1.00)	0.058	0.99 (0.98–1.00)	0.061
Antiviral treatment				
Oseltamivir	Reference	–	Reference	–
Baloxavir Marboxil	1.35 (1.12–1.64)	0.002	1.95 (1.49–2.55)	<0.001
Analgesic-antipyretic agents				
No	Reference	–	Reference	–
Yes	0.95 (0.75–1.19)	0.634	0.98 (0.73–1.35)	0.946
Antitussive/ expectorant agents				
No	Reference	–	Reference	–
Yes	1.22 (0.99–1.49)	0.062	1.26 (0.95–1.68)	0.115
Vaccination status				
No	Reference	–	Reference	–
Yes	1.07 (0.47–2.40)	0.878	0.75 (0.27–2.12)	0.592
Time from symptom onset to antiviral drugs (h)				
≤12	Reference	–	Reference	–
>12 and ≤ 24	0.95 (0.72–1.24)	0.683	0.86 (0.56–1.32)	0.489
>24 and ≤ 36	1.04 (0.80–1.35)	0.781	0.92 (0.62–1.37)	0.680
>36 and ≤ 48	0.89 (0.64–1.25)	0.505	0.84 (0.51–1.37)	0.479

TTAIS, time from treatment to alleviation of influenza symptoms; TTAF, time from treatment to alleviation of fever.

**Figure 3:**
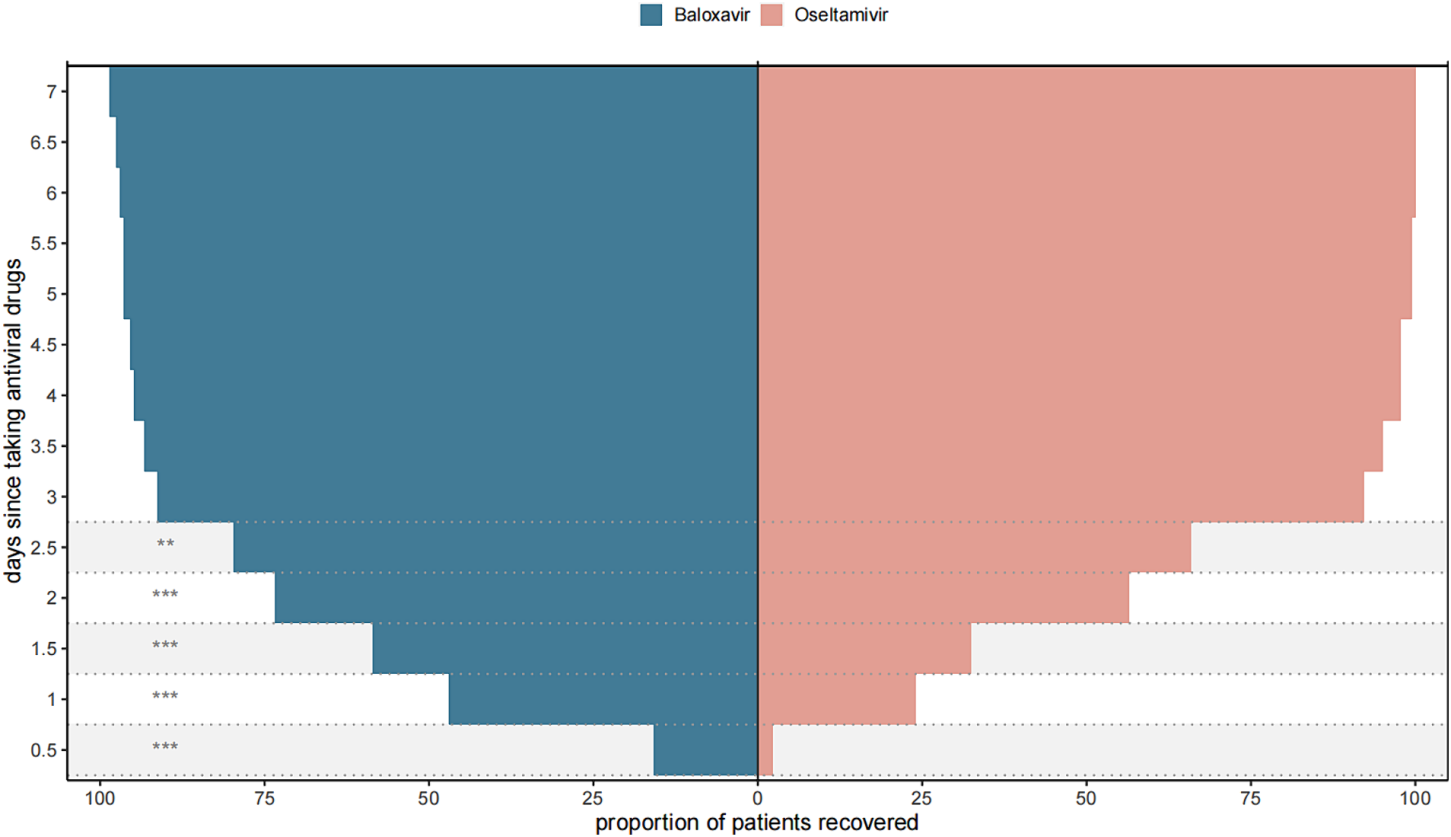
Proportion of patients in the Baloxavir Marboxil group and the Oseltamivir group who recovered after taking antiviral drugs. ^***^p < 0.001, ^**^p < 0.01, ^*^p < 0.05.

**Figure 4:**
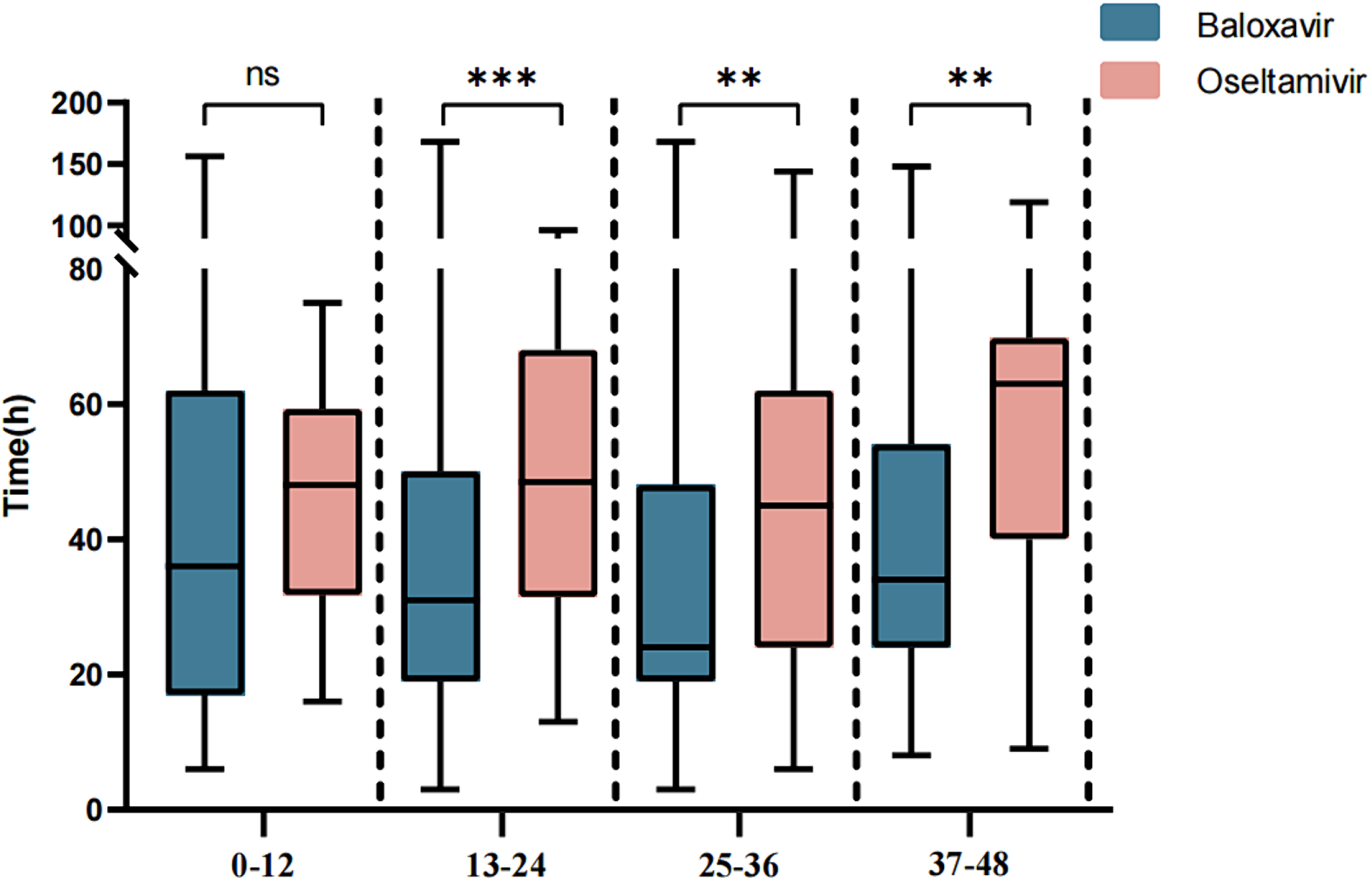
Comparison of the TTAIS between the Baloxavir Marboxil group and the Oseltamivir group after grouping according to symptom onset to time of taking antiviral drugs.

Next, we further evaluated the improvement ratio of influenza symptoms over time after taking medication. Within 2.5 days of medication, the proportion of influenza symptom improvement in the Baloxavir Marboxil group was significantly higher than that in the Oseltamivir group. However, there was no significant difference in the proportion of influenza symptom improvement between the two groups within 3–7 days of medication (Figure 3). We also assessed the relationship between the time from symptom onset to medication and the duration of symptoms after medication. When antiviral drugs were taken within 12 h of symptom onset, there was no significant difference in the median TTAIS between the Baloxavir Marboxil group and the Oseltamivir group (36 h [18–62] and 48.0 h [32.25–57.75], *p* = 0.1187). However, when antiviral drugs were taken within 12–48 h after symptom onset, the TTAIS in the Baloxavir Marboxil group was significantly shorter than that in the Oseltamivir group (Figure 4), which indicates that Baloxavir Marboxil was effective up to 48 h after symptom onset, but the effectiveness of Oseltamivir would significantly decrease after 12 h of symptom onset.

Finally, we evaluated the safety of Baloxavir Marboxil and Oseltamivir. In the Baloxavir Marboxil group, there were 3 cases of adverse events, including 1 case of bronchitis, 1 case of rash, and 1 case of asthma. The incidence rate of adverse events was 0.91%. Complications were mild in all 3 cases, and all eventually improved. No adverse events were observed in the Oseltamivir group. There was no significant difference in the incidence of adverse events between the two groups (*p* = 0.555) (Table 3). After excluding participants who had other influenza patients in their households before enrollment, the incidence rate of household influenza transmission during the 7-day follow-up period was 3.75% (11/293) in the Baloxavir Marboxil group and 4.72% (7/148) in the Oseltamivir group, with no significant difference between them (*p* = 0.625) (Table 3).

**Table 3:** Incidence of adverse events and household influenza transmission.

	Baloxavir Marboxil *n* = 330	Oseltamivir *n* = 179	*p* value
Adverse event			
Any event	3/330 (0.91)	0/330 (0)	0.555
Bronchitis	1/330 (0.30)	0/330 (0)	1.000
Rash	1/330 (0.30)	0/330 (0)	1.000
Asthma	1/330 (0.30)	0/330 (0)	1.000
Death	0/330 (0)	0/330 (0)	NA
Other suspected influenza or confirmed influenza	11/293 (3.75)	7/148 (4.72)	0.625

## Discussion

This is the first study to evaluate the efficacy and safety of Baloxavir Marboxil in outpatient non-severe influenza patients in the real world. Baloxavir Marboxil was approved for sale in China through the non-clinical pathway in 2021, and there is currently a lack of data on its use in the Chinese population. We aim to comprehensively evaluate the effectiveness and safety of Baloxavir Marboxil in non-severe influenza patients in China, by comparing it with the most used influenza drug, Oseltamivir, in fever clinic. This research will provide better guidance for the use of antiviral drugs in clinical settings.

The main purpose of this study is to investigate the effectiveness of Baloxavir Marboxil in improving influenza symptoms in the real world. In this study, compared to Oseltamivir, the use of Baloxavir Marboxil within 48 h of symptom onset resulted in faster symptom improvement in uncomplicated influenza outpatients. Previous studies have found that within 48 h of taking the medication, Baloxavir Marboxil can reduce upper respiratory viral load faster than Oseltamivir (Hayden et al., 2018; Ison et al., 2018; Baker et al., 2020). Higher viral load in influenza patients is closely associated with adverse outcomes, severe clinical symptoms, and prolonged hospital length of stay (Blanchon et al., 2013; Clark et al., 2016; Pereira et al., 2024). Therefore, faster reduction of viral load may be the reason why Baloxavir Marboxil can rapidly improve influenza symptoms (Hayden et al., 2018; Ison et al., 2018; Baker et al., 2020). However, it is worth noting that in a meta-analysis that included 3,771 outpatient influenza patients, although Baloxavir Marboxil was associated with a shorter duration of symptoms compared to Oseltamivir, there was no significant difference between the two (Kuo et al., 2021). In response to the mismatch between the reduction in viral load and the time to symptom alleviation, Frederick G et al. suggested that it may be due to the limited efficacy of antiviral drugs against influenza symptoms, and illness pathogenesis is linked to host proinflammatory responses (Hayden et al., 2018). However, in our study, combining anti-inflammatory drugs such as ibuprofen and acetaminophen did not significantly reduce symptom duration in patients compared to antiviral drugs alone, so further research is needed to explore the relationship between viral load and duration of symptom.

Fukao and colleagues found that in a mouse model of influenza A virus infection, delayed treatment (96 h postinfection) of Baloxavir Marboxil substantially reduced viral load, inflammatory response and mortality, whereas the effect was not significant with Oseltamivir (Fukao et al., 2019), consistent with our study. We found that the advantage of Baloxavir Marboxil in improving influenza symptoms relative to Oseltamivir was mainly seen in patients who did not seek timely medical attention (more than 12 h between the onset of symptoms and the time of administration of the medication), which may be related to the mechanism of the two drugs. Oseltamivir prevents the release of viral particles from the cell surface by blocking neuraminidase activity and does not prevent viral replication within the cell. In a study that included 955 patients with influenza, the use of Oseltamivir within 12 h of symptom onset shortened the duration of symptoms by 3.1 days compared to the use of Oseltamivir around 48 h (Aoki et al., 2003), underscoring the greater effectiveness of Oseltamivir when symptoms manifest earlier. As a viral mRNA polymerase inhibitor, Baloxavir Marboxil can block intracellular viral replication by inhibiting mRNA synthesis (Dias et al., 2009; Genentech, 2023), so the window of time in which Baloxavir Marboxil can be effective in improving the duration of symptoms may be longer compared to Oseltamivir.

Differences in antiviral mechanisms enable the combination of Baloxavir Marboxil and Oseltamivir for influenza treatment. Animal studies demonstrated that combining Baloxavir Marboxil with Oseltamivir effectively reduces mortality in animal models and mitigates influenza virus resistance better than monotherapy (Park et al., 2021; Koszalka et al., 2022). In a randomized, double-blind clinical trial, combining Baloxavir Marboxil with a neuraminidase inhibitor (NAI) (oseltamivir in the majority of cases) for hospitalized influenza patients did not markedly shorten the median time to clinical improvement compared to NAI alone. However, combining antivirals could decrease viral load and reduce emergence of drug resistance compared with NAI alone. This suggests the combination’s potential significance for certain populations (e.g., immunosuppressed individuals) and in preventing nosocomial transmission, warranting further investigation (Kumar et al., 2022).

In our study, the incidence of adverse events in the Baloxavir Marboxil group was 0.91%, whereas in previous studies, the incidence of Baloxavir Marboxil-related adverse events ranged from 1.9 to 6%. The low incidence of adverse events in our study may be due to several factors: first, our study did not restrict the use of symptomatic drugs by the subjects, which may have affected the occurrence of adverse events. Second, some of the data in our study were derived from retrospective surveys, and mild adverse events are easily overlooked and underreported by the subjects, leading to an underestimation of adverse events incidence. However, overall, there was no significant difference in the incidence of adverse events between Baloxavir Marboxil and Oseltamivir in our study, indicating that Baloxavir Marboxil has good safety, similar to previous studies.

In comparison with Oseltamivir, we did not find advantage of Baloxavir Marboxil in preventing household transmission of the influenza. In a real-world study based on the Health Insurance Claims Database in Japan, although its primary outcome indicated significant transmission reduction potential of Baloxavir Marboxil, there was no difference in household transmission rates between the Baloxavir Marboxil group and the Oseltamivir group in individuals over the age of 12, which is similar to our study (Komeda et al., 2021b). However, it is worth noting that in another study based on the same database, after using a different model that considered patients’ viral shedding period and effectively excluded household infections from external sources, Baloxavir Marboxil was found to be more effective in reducing household transmission rates compared to Oseltamivir (Miyazawa et al., 2022). In summary, the transmission rate of household infection was influenced by many factors, such as the home environment, susceptibility of contacts to influenza, hygienic condition, etc., so more research is needed in the future to determine whether Baloxavir Marboxil can reduce household transmission of the influenza in Chinese family.

There are several limitations to this study. Firstly, the participating centers in this study are all from the East China region, and all subjects were infected with influenza A virus, which limits the generalizability of this cohort. Therefore, in the future, more regions and other types of influenza virus patients need to be included. Secondly, the time for symptom relief in this study was self-reported by patients during telephone follow-ups and was not objectively assessed, so there may be some bias. Furthermore, due to the limitations of real-world studies, we did not measure the patients’ viral load or plasma drug concentrations. This restricted our ability to further analyze the relationship between symptom relief and viral load, as well as the differences in drug metabolism among different groups. Finally, when evaluating household transmission of the influenza, this study did not consider factors such as the number of cohabiting household members and external transmission, so further research is needed to investigate the role of Baloxavir Marboxil in preventing household transmission of the influenza in China.

## Conclusion

Baloxavir Marboxil significantly reduces the duration of symptoms in patients with uncomplicated influenza compared with Oseltamivir. No evident safety concerns were observed in patients taking Baloxavir Marboxil. Our findings suggested that compared to Oseltamivir, Baloxavir Marboxil might be more appropriate for patients with influenza 12–48 h after symptom onset.

## Data availability statement

The original contributions presented in the study are included in the article/Supplementary material, further inquiries can be directed to the corresponding authors.

## Ethics statement

The revised Ethics statement is as follows: The studies involving humans were approved by institutional review board from Huashan Hospital (KY2022-888). The studies were conducted in accordance with the local legislation and institutional requirements. Oral informed consent for participation in this study was provided by the participants’ legal guardians/next of kin.

## Author contributions

JC: Visualization, Software, Project administration, Methodology, Formal analysis, Data curation, Writing – original draft. HW: Visualization, Software, Methodology, Formal analysis, Data curation, Writing – original draft. XY: Writing – review & editing, Project administration, Data curation. SL: Writing – review & editing, Project administration, Data curation. ZT: Writing – review & editing, Project administration, Data curation. ZL: Writing – review & editing, Project administration, Data curation. DL: Writing – review & editing, Data curation. JQ: Writing – review & editing, Data curation. XL: Writing – review & editing, Data curation. JiaW: Writing – review & editing, Data curation. JieW: Writing – review & editing, Data curation. JA: Writing – review & editing, Supervision, Data curation, Conceptualization. YP: Writing – review & editing, Supervision, Data curation, Conceptualization. LQ: Writing – review & editing, Supervision, Data curation, Conceptualization. SW: Writing – review & editing, Supervision, Funding acquisition, Conceptualization.
