# Supplementary material for: Real-world effectiveness and safety of Baloxavir Marboxil or Oseltamivir in outpatients with uncomplicated influenza A: an ambispective, observational, multi-center study
Source: Front Microbiol. 2024 Jul 23;15:1428095. doi: 10.3389/fmicb.2024.1428095 (PMC11300241; doi:10.3389/fmicb.2024.1428095)
Supplement: Supplementary file 1 [file Data_Sheet_1.pdf]

Real-world effectiveness and safety of Baloxavir Marboxil or Oseltamivir in outpatients with uncomplicated influenza A: an ambispective, observational, multi-center study

Supplementary Material

Jianpeng Cai, Hongyu Wang, Xiaoting Ye, Shengjia Lu, Zhili Tan, Zhonghua Li, Dan Lin, Jiancheng Qian, Xiaoxian Lu, Jiaolong Wan, Jie Wang, Jingwen Ai*, Yonglan Pu*, Lihong Qu*, Sen Wang *

*** Correspondence:**
Jingwen Ai:
jingwenai1990@126.com

Yonglan Pu:
tcyypyl@163.com

Lihong Qu:
18916510601@163.com

Sen Wang:

senwang@fudan.edu.cn

Supplementary Table 1. Demographic and clinical characteristic of participants in retrospective study or prospective study.

Characteristics	Retrospective n=118	Prospective n=391	*P value*
**Gender, n(%)**			0.172
Male	67 (56.78)	194 (49.62)	
Female	51 (43.22)	197 (50.38)	
**Age, mean ± SD [Median, IQR] (years)**	33.97 ±13.18 [31, 25-40]	37.48±14.86 [36, 26-45]	0.02
**Time from symptom onset to antiviral drugs, n(%)**			<0.001
≤12 hr	16 (13.56)	67 (17.14)	
>12 to ≤ 24hr	32 (27.12)	121 (30.95)	

>24 to ≤ 36hr	67 (56.78)	145 (37.08)	
>36 to ≤ 48hr	3 (2.54)	58 (14.83)	
**Influenza vaccination, n (%)**			0.344
Yes	0 (0)	6 (1.53)	
No	118 (100)	385 (98.47)	
**Comorbidities, n (%)**			
Hypertension	3 (2.54)	20 (5.12)	0.316
Diabetes	2 (1.69)	8 (2.05)	0.810
Cardiovascular disease (except Hypertension)	0 (0)	8 (2.05)	0.208
Endocrine diseases (except Diabetes)	1 (0.85)	4 (1.02)	0.865
Lung disease	0 (0)	4 (1.02)	0.578
Hepatic disease	0 (0)	4 (1.02)	0.578
Hematologic disease	0 (0)	2 (0.51)	1.000
Cancer	0 (0)	1 (0.26)	1.000
**High risk of developing influenza-associated complications, n (%)**	7 (5.93)	43 (11.00)	
**Influenza symptoms, n (%)**			
Fever	67 (56.78)	208 (53.20)	0.494
Cough	60 (50.85)	307 (78.52)	<0.001
Sore throat	66 (55.93)	217 (55.50)	0.934
Nasal congestion or runny nose	38 (32.20)	127 (32.48)	0.955
Chills	14 (11.86)	133 (34.02)	<0.001
Headache	41 (34.75)	196 (50.13)	0.003
Muscle or joint pain	38 (32.20)	220 (56.27)	<0.001
Fatigue	58 (49.15)	130 (33.25)	0.002
**Antiviral treatment**			0.099
Baloxavir Marboxil	84 (71.19)	246 (62.92)	
Oseltamivir	34 (28.81)	145 (37.08)	
**Analgesic-antipyretic**	102 (86.44)	262 (67.01)	<0.001
**Antitussive and expectorant agents**	50 (42.37)	221 (56.52)	0.007
**Duration of symptoms, mean ± SD [Median, IQR] (hour)**	43.12±33.60 [24, 24-48]	42.82±29.00 [39, 20-63]	0.731
**Duration of fever, mean ± SD [Median, IQR] (hour)**	27.89±18.17 [24, 18-24]	25.11±21.21 [19, 10-35]	0.232

Supplementary Figure Legends

Figure S1. Kaplan–Meier analysis of the duration of each influenza symptom after taking antiviral drugs in the Baloxavir Marboxil group and the Oseltamivir group. (A) cough (B) sore throat (C) nasal congestion or runny nose (D) chills (E) headache (F) muscle or joint pain (G) fatigue.

Figure S2. Kaplan–Meier analysis of the duration of each influenza symptom after taking antiviral drugs in the Baloxavir Marboxil group and the Oseltamivir group after 1:1 matched. (A) cough (B) sore throat (C) nasal congestion or runny nose (D) chills (E) headache (F) muscle or joint pain (G) fatigue.

Figure S3. Duration of symptoms with or without symptomatic treatment. (A) TTAIS (B) TTAF.
