# Supplementary figures and images for: Real-world effectiveness and safety of Baloxavir Marboxil or Oseltamivir in outpatients with uncomplicated influenza A: an ambispective, observational, multi-center study
Source: Front Microbiol. 2024 Jul 23;15:1428095. doi: 10.3389/fmicb.2024.1428095 (PMC11300241; doi:10.3389/fmicb.2024.1428095)

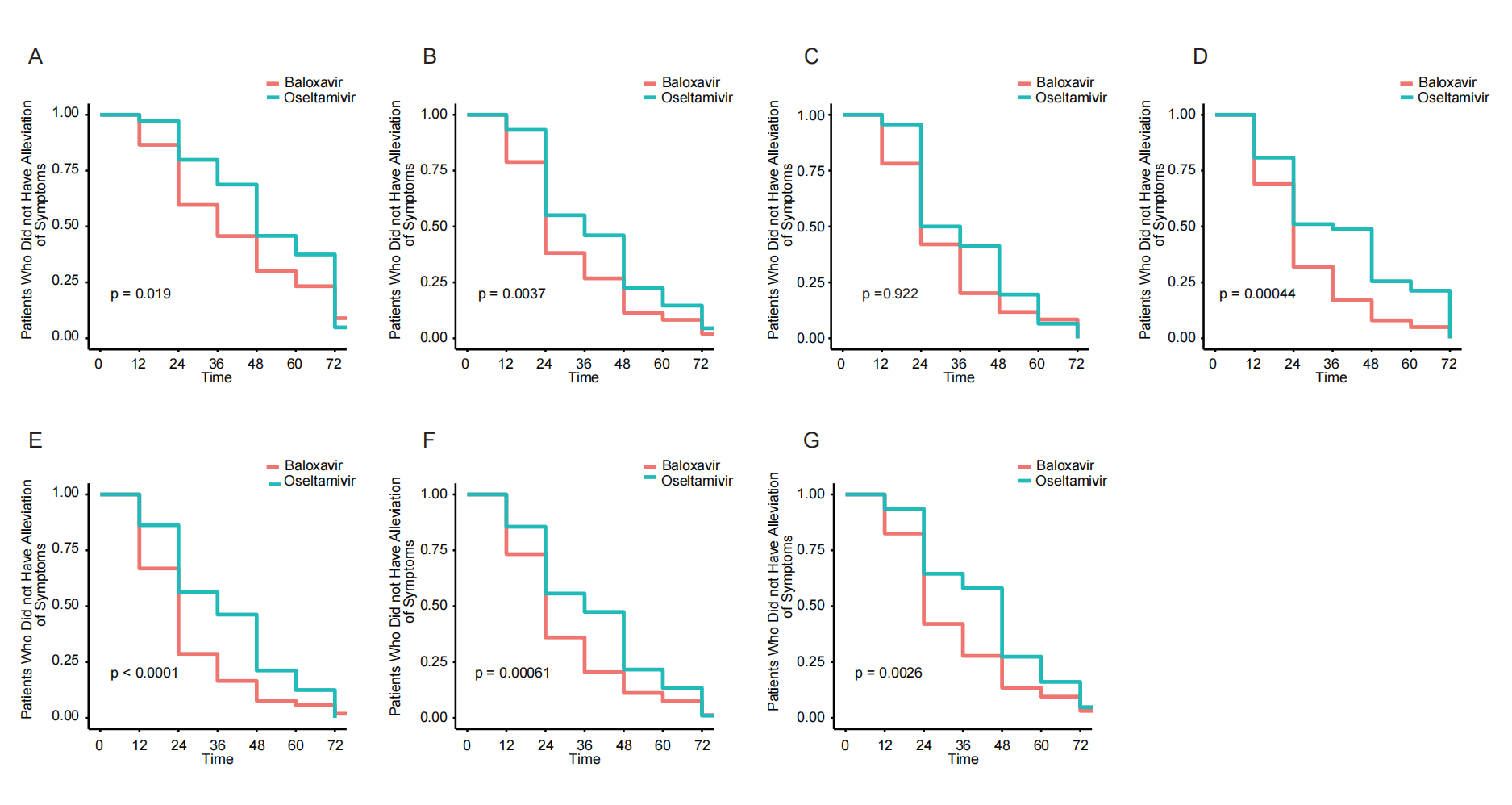

Supplement: Supplementary file 2 [file Image_1.TIF]

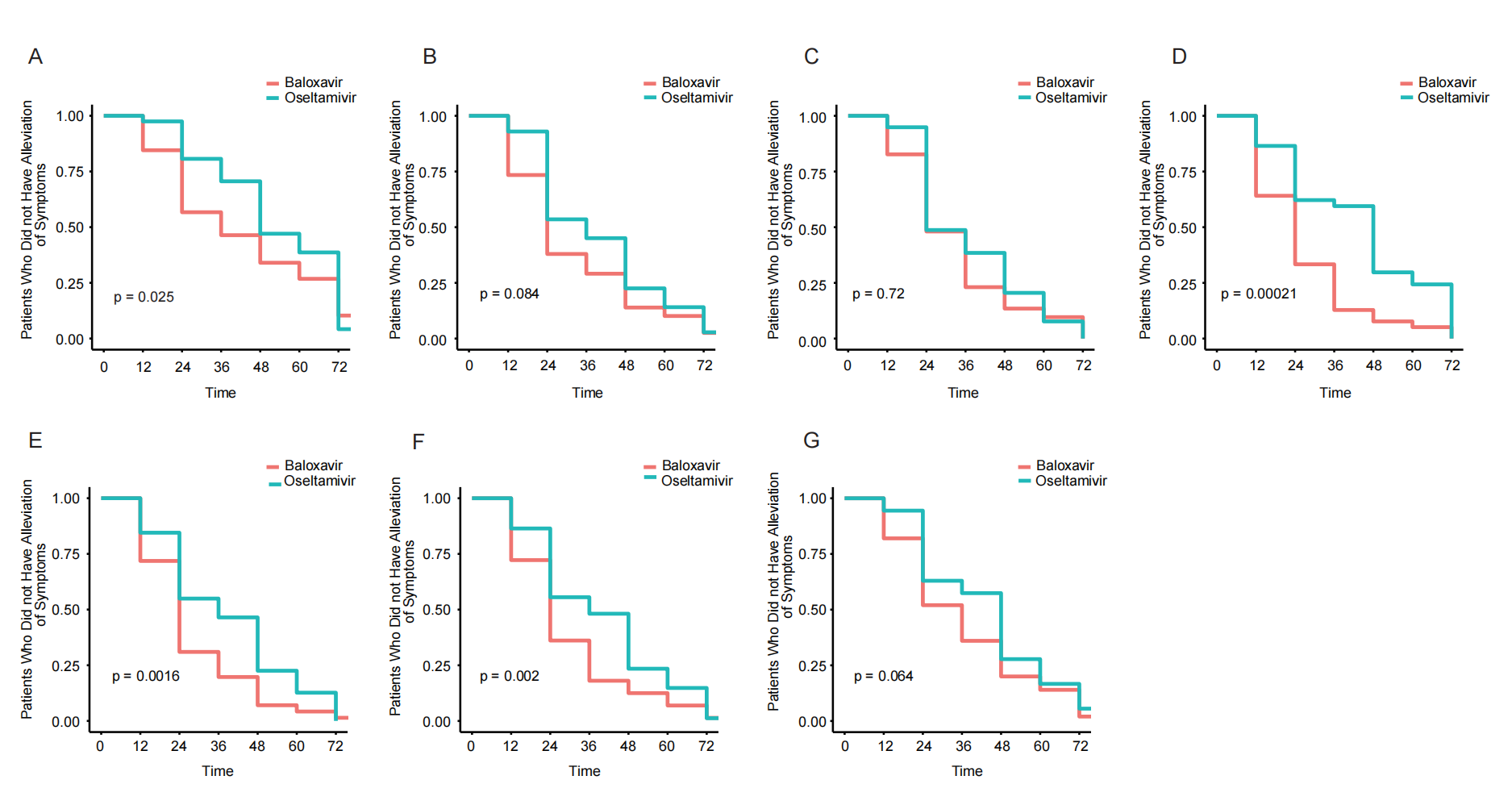

Supplement: Supplementary file 3 [file Image_2.TIF]

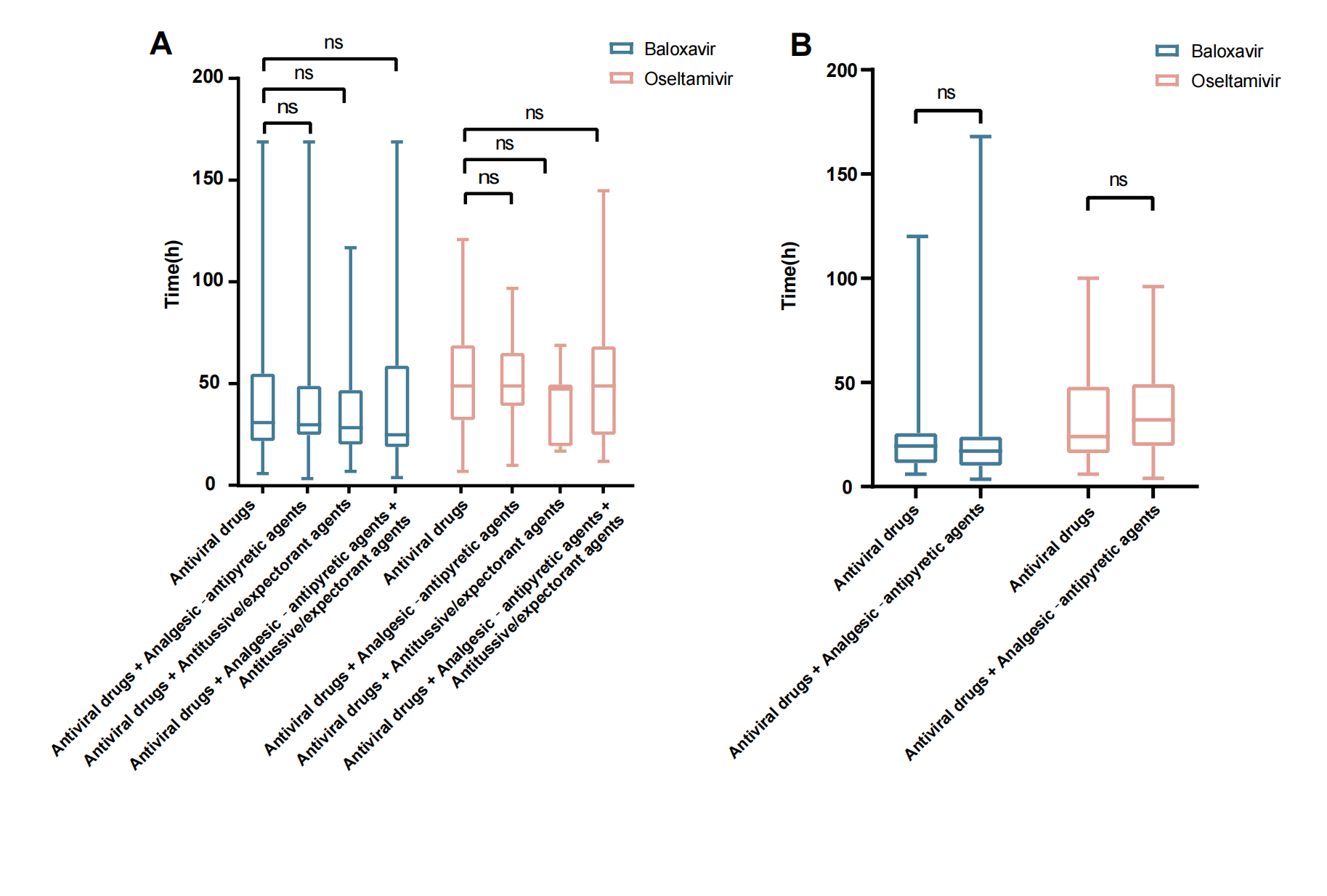

Supplement: Supplementary file 4 [file Image_3.TIF]
